# Foliar application of methyl jasmonate affects impatiens walleriana growth and leaf physiology under drought stress

**DOI:** 10.1080/15592324.2023.2219936

**Published:** 2023-06-08

**Authors:** Marija Đurić, Angelina Subotić, Ljiljana Prokić, Milana Trifunović-Momčilov, Snežana Milošević

**Affiliations:** a Department for Plant Physiology, Institute for Biological Research “Siniša Stanković”, National Institute of Republic of Serbia, University of Belgrade, Belgrade, Serbia; b Faculty of Agriculture, University of Belgrade, Belgrade, Serbia

**Keywords:** horticultural plants, abiotic stress, soil water content, elicitation, leaf physiology

## Abstract

In this study, the effects of foliar applied methyl jasmonate (MeJA) on drought-stressed *Impatiens walleriana* growth and leaf physiology parameters: stomatal conductance, chlorophyll, flavonoid, anthocyanin, and nitrogen balance index (NBI), were evaluated. These parameters could serve as indicators of drought tolerance of *I. walleriana*, a popular horticultural plant worldwide that is very sensitive to drought. The experiment included four treatments: control, drought-stressed plants sprayed with distilled water, drought-stressed plants sprayed with 5 µM MeJA, and drought-stressed plants sprayed with 50 µM MeJA. Foliar spraying with MeJA was performed twice: seven days before and on the day of drought induction. The stressed plant groups were non-irrigated to achieve soil water contents (SWC) of 15 and 5%, while control plants were well-watered throughout the experiment (35–37% SWC). The results of this study showed that drought significantly reduced *I. walleriana* fresh and dry shoot weight, as well as total leaf area, but did not impact on dry matter content. The foliar application of MeJA improved growth parameters of *I. walleriana*, depending on the elicitor concentration and drought intensity. Stomatal conductance was slightly reduced at 5% SWC, and foliar applied MeJA at both concentrations. The flavonoid index was slightly reduced at 15 and 5% SWC when 50 µM MeJA was foliar applied, while there were no observed changes in the anthocyanin index in any treatments. The foliar application of 50 µM MeJA increased the chlorophyll index and NBI of *I. walleriana* at 5% SWC, indicating a contribution of the elicitor to plant drought tolerance at the physiological level.

## 1. Introduction

*Impatiens walleriana* is one of the most popular species in the genus *Impatiens*, with fleshy, succulent leaves, and variously colored flowers from early spring to late fall^1^. Flower colors can be red, white, orange, purple, and pink. It is one of the three species (together with *I. hawkeri* and *I. balsamina*) of the genus *Impatiens* that have been commercially produced in Serbia for many years. Due to its decorative properties and long flowering period, *I. walleriana* has been one of the most popular horticultural species worldwide since the 19^th^ century^2^, but importance of this horticultural plant is not exclusively related to its ornamental properties^3–7^. This plant has high requirements for the presence of water in the substrate, the absence of which leads to a rapid drop in turgor pressure in cells and tissue dehydration. Insufficient water availability during plant production can affect growth and affect the decorative properties of the plants. In addition, lack of water during transport of plants to markets can have significant negative effects on plant quality.

Water deficit, namely drought in general, is an abiotic stress factor that can severely affect plant growth and development. Photosynthesis is a key factor for plant biomass and can be significantly reduced during drought due to the stomatal closure and reduced gas exchange^8^. In addition, exposure of plants to drought leads to the production of reactive oxygen species (ROS), which in turn activate many signal cascades leading to the synthesis of antioxidant compounds^8^. Changes in photosynthetic pigment compositions, such as chlorophyll content, are closely related to the photosynthesis efficiency^9^, while secondary metabolites, such as polyphenols, play an important role in plant antioxidant defense^10,11^. Nitrogen balance index (NBI) is the ratio between chlorophyll and flavonoids, and under low nitrogen (N) availability, plants allocate excess carbon to the synthesis of polyphenols^12^. Therefore, polyphenol content can also be used as another potential indicator of the N status of plants. As a constituent of amino acids, nucleotides, chlorophyll, and many other metabolites, the importance of N is undoubtedly crucial for many physiological processes. Since more than 50% of leaf N is stored in chloroplasts and used for the synthesis of photosynthetic components, it could be concluded that leaf photosynthetic capacity is closely and positively correlated with N content^13^.

Improving abiotic stress tolerance through the exogenous application of different compounds called elicitors, is a common strategy in research. Elicitors are low molecular weight compounds and can be divided into two groups based on their origin: abiotic and biotic^14,15^. Abiotic elicitors include physical (high or low temperatures, UV radiation, drought, salinity), chemical (heavy metals, CaCl_2_, CuCl_2_, chitosan), and hormonal elicitors (plant growth regulators). In contrast, biotic elicitors are substances with biological origin and include polysaccharides from the cell wall of plants (pectin, chitin, cellulose), various microorganisms, and plant growth-promoting *Rhizobacteria* ^15,16^. Both groups of elicitors variously affect plant growth and development, as well as the ability to trigger adaptive responses that can alleviate the harmful consequences of certain abiotic or biotic stress factors. Synthetic plant hormones, known as plant growth regulators, are commonly used for the triggering process by spraying on leaves, adding to the *in vitro* culture medium, or seeds priming by dipping them in elicitor solutions. Elicitation with plant growth regulators causes physiological alterations in plants that can improve their ability to respond to abiotic stresses. In general, the plant response to stress depends on the applied elicitor concentration, duration of application, ontogenetic phases, and genotype. The most commonly used plant growth regulators as elicitors are abscisic acid, salicylic acid, jasmonate, and brassinosteroids^15–20^. Several publications have indicated that elicitation with a jasmonate methylated derivative – methyl jasmonate (MeJA) can neutralize the negative effects of drought on plant growth and development. The foliar application of MeJA in different plant species increased drought resistance^21–25^. Growth-stimulating effect of MeJA at a concentration of 5 µM has been recently described for *I. walleriana* grown *in vitro* ^26^. However, it is unknown whether MeJA can reduce water stress in *ex vitro*-grown *I. walleriana*. Additionally, previous research indicated a negative effect of drought induced by polyethylene glycol *in vitro* on the growth and development of *I. walleriana*, as well as a positive effect of exogenous application of salicylic acid in neutralizing the effects of drought *in vitro* and *ex vitro* ^1,27,28^. Also, drought in the growth chamber affects the growth, total polyphenol content, antioxidant status, abscisic acid metabolic and aquaporin gene expression in *I. walleriana* ^29,30^.

In this work, the effects of foliar applied MeJA in different concentrations on *I. walleriana* growth and physiology under drought were investigated. The main focus was on changes in stomatal conductance and parameters measured by Dualex: chlorophyll, flavonoid, and anthocyanin indexes, as well as the NBI. Among numerous traits of the *I. walleriana*, the physicochemical components of the leaves play an important role in evaluating the plant drought tolerance. Therefore, in this study, we investigated the effects of drought and MeJA on the indicators of leaf photosynthetic capacity, and provided new information on the physiological mechanisms underlying the response of *I. walleriana* to drought.

## 2. Material and methods

### 2.1. Plant material and experiment design

Seeds of *I. walleriana*, Xtreme Scarlet variety (Syngenta) were used as the starting material for the experimental work. Seeds were germinated in May 2021 in a growth chamber at the Faculty of Agriculture, University of Belgrade, in plates containing commercial substrate (Klasman Potgrond H) (Hidroponika). Klasman Potgrond H substrate is used for growing, planting, and transplanting various types of plants. It contains black and white sphagnum peat, microelements, and a dose of NPK fertilizer. Physical conditions during seed germination require 100% relative humidity and a temperature between 22°C and 25°C. At temperatures below 22°C, dormancy occurs, whereas seed germination is significantly slowed at temperatures above 25°C. Relative humidity of 100% was achieved by covering the plates with aluminum foil after sowing and watering the seeds. The aluminum foil was removed from the plates after seed germination. After one month of growth in the plates, plants were transplanted into pots of 10 × 10×13 cm, containing 450 g of Klasman Potgrond H substrate. The physical conditions for further cultivation of the plants included temperatures in the range from 22 to 26°C and relative humidity in the range from 30 to 60%, photoperiod of 14 h and illuminance of 250 mmol m^−1^ s^−1^.

**Figure 1.**
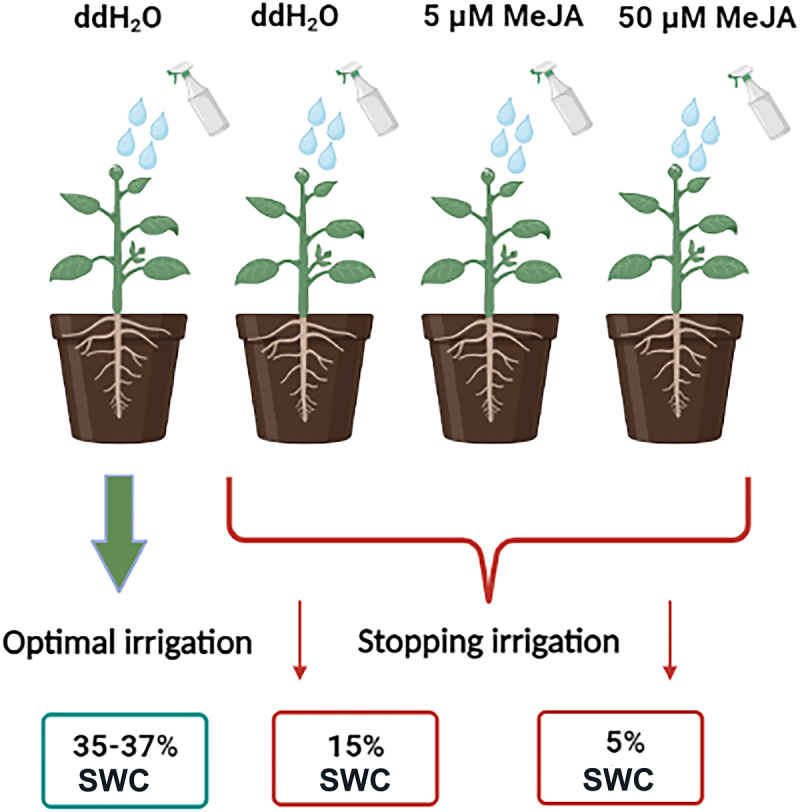
Schematic illustration of experiment design with foliar applied elicitors in drought-stressed I. walleriana. SWC – soil water content; MeJA – methyl jasmonate.

Exogenous application of MeJA (Sigma Aldrich, Sent Luis, Misuri, USA) was performed using two different concentrations (5 and 50 µM), on plants exposed to drought (15 and 5% SWC). The stock solution of MeJA (1 M) was prepared by dissolving 96% ethanol with the addition of a few drops of Tween 20, whose detergent properties allow better retention and thus absorption of the solution by leaves. Further dilutions of MeJA stock solution were made using distilled water (ddH_2_O). Spraying the plants with MeJA solution was performed twice during the experimental work. The first spraying was performed seven days before the drought stress imposition, and the second was performed on the day of drought stress imposition. Depending on the treatment, 20 ml of a MeJA solution at 5 and 50 μM concentrations were sprayed on each plant. Since 96% ethanol was used to dissolve MeJA, ethanol, and Tween 20, were added to the distilled water and both control plants and a group of drought-stressed plants were sprayed. On the day of drought induction, plants were 44 days old, as in the previously conducted experiment^29^. Drought stress was imposed by stopping irrigation to obtain 15 and 5% soil water content (SWC), while the control plants were well watered during the entire experiment duration (from 35 to 37% SWC). Nine days without a water supply were required to reach 15% SWC, while 20 days were required to reach 5% SWC, as described previously by Đurić et al.,^29^. Physiological measurements were performed at the moment when soil moisture reached 15 and 5%. The experiment had four treatments: control plants treated with ddH_2_O (C), drought-stressed plants treated with ddH_2_O (D), drought-stressed plants treated with 5 µM MeJA (D +5 µM MeJA), and drought-stressed plants treated with 50 µM MeJA (D +50 µM MeJA), with three replications and four plants in each (*n* = 12). A schematic representation of the experimental design is presented in Figure 1.

### 2.2. Soil water content measurement

Percent soil water content (SWC) was measured daily in the morning (9 am) using a theta probe (type ML2×, Delta-T Devices Ltd, Cambridge, U.K.).

### 2.3. Growth parameters

Growth parameters included measurement of the fresh weight (FW) of shoots, total leaf area (*LI-3100 AREA METER, LI.COR. Inc. Lincoln, NE, USA*), and subsequently measuring the dry weight (DW) of shoots. The DW of shoots was obtained by drying the plant material for several days at room temperature and then for 48 h at 70°C. The percentage of dry matter content (DMC) was calculated as DW per unit of FW of above-ground parts^31^.

### 2.4. Stomatal conductance and dualex measurements

Stomatal conductance was measured using a porometer (*AP4 Porometer, Delta T Devices, Cambridge UK*), after measuring SWC in the morning. Leaf Chlorophyll, Flavonoid, Anthocyanin, and NBI were measured using a Dualex (*FORCE-A, Orsay, France*) an optical sensor that provides rapid, simple and nondestructive measurements of plant leaves^32^. For these measurements, third or fourth leaf from the plant apex is usually used, depending on their area and devices sensitivity.

### 2.5. Statistical analysis

Statistical differences between experimental treatments were assessed by ANOVA using the STATISTICA software (version 8), and the results are expressed as means ± standard error (SE). The mean differences between three replications per treatment were compared by the least significant difference (LSD) method with a statistical significance of *p* ≤ 0.05. Graphical representation of results was performed using the Microsoft Office Excel program (2010), while for schematic representation of Material and Methods Biorender program was used (https://biorender.com/).

## 3. Results

### 3.1. *Changes in growth parameters of* I. walleriana *after foliar application of MeJA during drought*

**Figure 2.**
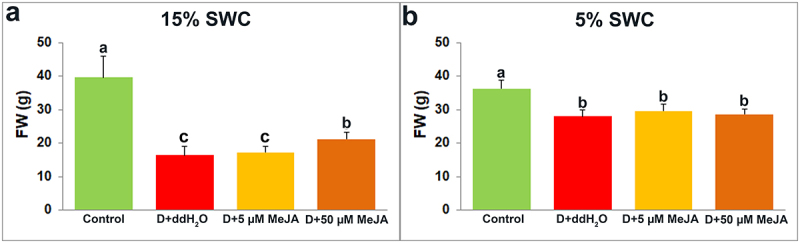
The effect of foliar applied MeJA on I. walleriana shoots FW at 15 (A) and 5% (B) SWC. SWC – soil water content; FW – fresh weight. Results are presented as mean ± SE, with significant differences between treatments based on LSD test (*p* ≤ 0.05).

The effect of foliar applied MeJA (5 and 50 µM) on *I. walleriana* shoots FW under 15 and 5% SWC is shown in Figure 2A,B. Compared to control shoots, drought significantly reduced FW by 58.50 and 22.80% at 15 and 5% SWC, respectively. Foliar application of 50 µM MeJA increased shoots FW by 28.85% under 15% SWC, compared with drought-stressed shoots treated with ddH_2_O. Foliar applied MeJA had no significant effect on shoots FW at 5% SWC.

**Figure 3.**
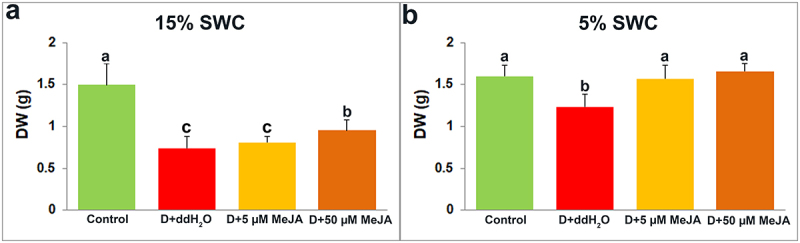
The effect of foliar applied MeJA on I. walleriana shoots DW at 15 (A) and 5% SWC (B). SWC – soil water content; DW – dry weight. Results are presented as mean ± SE, with significant differences between treatments based on LSD test (*p* ≤ 0.05).

Soil irrigation to 15% and 5% reduced *I. walleriana* shoots DW by 50% and 23% compared to control shoots (Figure 3A). However, foliar applied MeJA in concentration of 50 µM increased shoots DW by 28.85% compared with drought-stressed shoots treated with ddH_2_O at 15% SWC. At 5% SWC, both foliar applied MeJA concentrations (5 and 50 µM) increased shoots DW (by 27.33 and 34.80%, respectively), compared with drought-stressed shoots foliar sprayed with ddH_2_O (Figure 3B).

**Figure 4.**
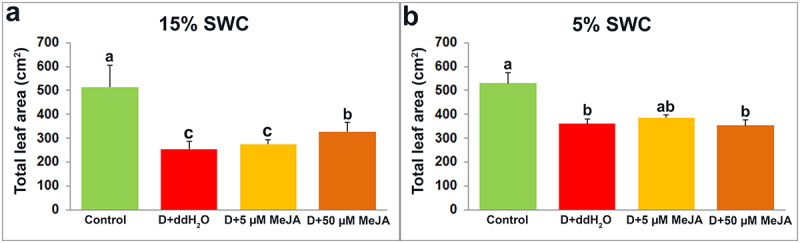
The effect of foliar applied MeJA on I. walleriana total leaf area at 15 (A) and 5% (B) SWC. SWC – soil water content. Results are presented as mean ± SE, with significant differences between treatments based on LSD test (*p* ≤ 0.05).

Compared to control shoots, total leaf area in drought-stressed plants was reduced by 50.31 and 31.80% under 15 and 5% SWC, respectively (Figure 4A,B). In plants foliar sprayed with 50 µM MeJA and irrigated to 15% SWC, total leaf area was significantly increased by 28.50%, compared with drought-stressed plants foliar sprayed with ddH_2_O (Figure 4A). On the other hand, at 5% SWC, foliar applied 5 µM MeJA only slightly increased the total leaf area compared with plant foliar sprayed with ddH_2_O (Figure 4B).

**Figure 5.**
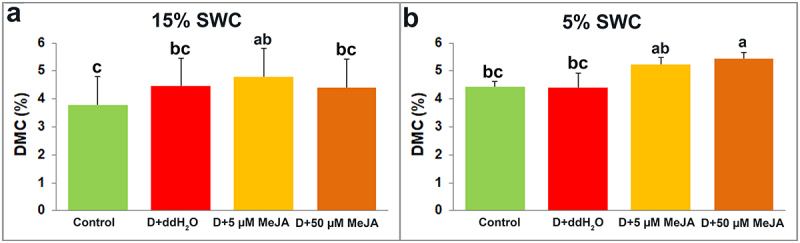
The effect of foliar applied MeJA on I. walleriana DMC at 15 (A) and 5% (B) SWC. SWC – soil water content; DMC – dry matter content. Results are presented as mean ± SE, with significant differences between treatments based on LSD test (*p* ≤ 0.05).

The changes in *I. walleriana* DMC are presented in Figure 5A,B. Compared to control plants, plants foliar sprayed with ddH_2_O, 5 and 50 µM MeJA increased their DMC for 17.86, 26.68, and 16.53%, respectively, at 15% SWC. At 5% SWC, only plants foliar sprayed with MeJA (5 and 50 µM), expressed increment in DMC for 18.00 and 22.38%, respectively, in comparison to control plants.

**Figure 6.**
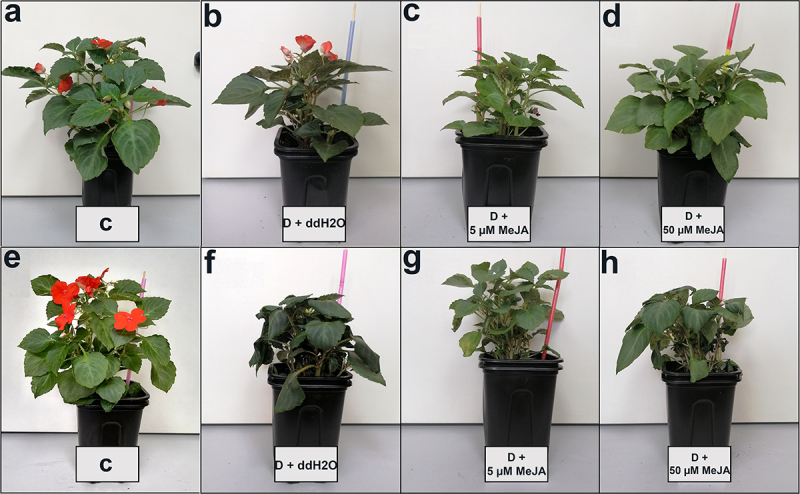
The effect of MeJA foliar application on the I. walleriana growth at 15% SWC: a – control, b – drought + ddH_2_O, c – drought + 5 µM MeJA and d – drought + 50 µM MeJA; and 5% SWC: e – control, f – drought + ddH_2_O, g – drought + 5 µM MeJA and h – drought + 50 µM MeJA. SWC – soil water content.

In Figure 6, morphological differences between *I. walleriana* during drought after foliar pretreatment with MeJA or ddH_2_O, are presented. It could be noticed that plants foliar sprayed with 50 µM MeJA grew better at 15% SWC (Figure 6D) and had higher total leaf area. Similarly, plants treated with 5 µM MeJA had a slightly higher total leaf area at 5% SWC (Figure 6G).

### 3.2. *Stomatal conductance in* I. walleriana *leaves foliar sprayed with MeJA during drought*

**Figure 7.**
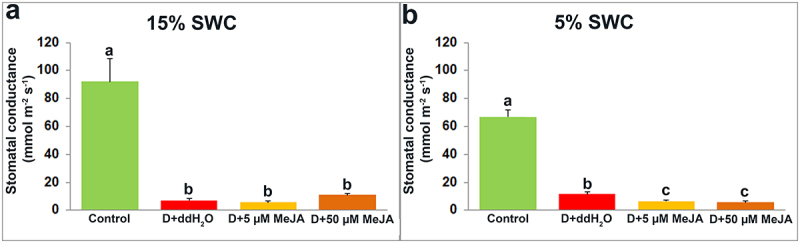
The effect of foliar applied MeJA on I. walleriana stomatal conductance at 15 (A) and 5% (B) SWC. SWC – soil water content. Results are presented as mean ± SE, with significant differences between treatments based on LSD test (*p* ≤ 0.05).

Based on the results obtained for the stomatal conductance measurement, it can be noticed that both drought treatments (15 and 5% SWC), significantly reduced stomatal conductance in plants foliar sprayed with ddH2O (by 92.20 and 82.75%, respectively), compared with control plants (Figure 7A,B). At 15% SWC, plants foliar sprayed with 50 µM MeJA showed slightly increment in stomatal conductance compared to drought-stressed plants foliar sprayed with ddH_2_O and 5 µM MeJA, but there was no significant difference between these two groups of plants (Figure 7A). Alternatively, at 5% SWC, plants treated with both MeJA concentrations had slightly reduced stomatal conductance compared with plants treated with ddH_2_O (Figure 7B) but without significant difference between MeJA treatments.

### 3.3. *Physicochemical composition of* I. walleriana *leaves foliar sprayed with MeJA during drought*

**Figure 8.**
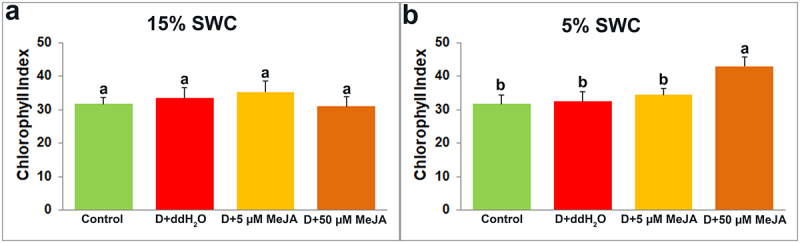
The effect of foliar applied MeJA on I. walleriana chlorophyll index at 15 (A) and 5% (B) SWC. SWC – soil water content. Results are presented as mean ± SE, with significant differences between treatments based on LSD test (*p* ≤ 0.05).

**Figure 9.**
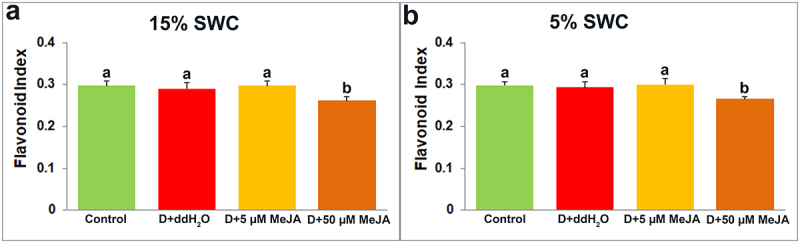
The effect of foliar applied MeJA on I. walleriana flavonoid index at 15 (A) and 5% (B) SWC. SWC – soil water content. Results are presented as mean ± SE, with significant differences between treatments based on LSD test (*p* ≤ 0.05).

**Figure 10.**
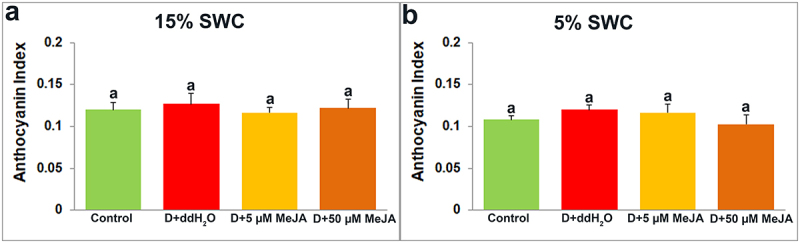
The effect of foliar applied MeJA on I. walleriana anthocyanin index at 15 (A) and 5% (B) SWC. SWC – soil water content. Results are presented as mean ± SE, with significant differences between treatments based on LSD test (*p* ≤ 0.05).

**Figure 11.**
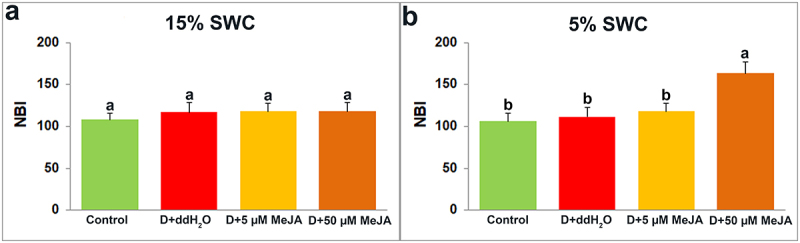
The effect of foliar applied MeJA on I. walleriana NBI at 15 (A) and 5% (B) SWC. SWC – soil water content; NBI – nitrogen balance index. Results are presented as mean ± SE, with significant differences between treatments based on LSD test (*p* ≤ 0.05).

Dualex measurement provides insight into four leaf indexes: Chlorophyll (Figure 8), Flavonoid (Figure 9), Anthocyanin (Figure 10), and Nitrogen (Figure 11).

Results showed no significant changes in the chlorophyll index at 15% SWC between treatments (Figure 8A). However, foliar applied 50 µM MeJA induced a significant increment of the chlorophyll index at 5% SWC, compared with control, as well as with drought-stressed plants foliar sprayed with ddH_2_O or 5 µM MeJA (Figure 8B).

The flavonoid index was reduced only in plants foliar sprayed with 50 µM MeJA at both drought stress intensities (Figure 9A,B), while the anthocyanin index was similar after all applied treatments (Figure 10A,B).

The NBI was increased only in plants foliar sprayed with a higher elicitor concentration (50 µM MeJA) at 5% SWC (Figure 11B). At 15% SWC, there were no significant differences in NBI between control and treated groups of *I. walleriana* plants (Figure 11A).

## 4. Discussion

In this study, the effect of drought and foliar applied MeJA on the morphological and physiological responses of *I. walleriana* was evaluated. Drought significantly reduced the FW and DW of *I. walleriana* shoots, and the total leaf area. Negative effects of drought on *ex vitro* grown *I. walleriana* were previously described by Đurić et al.,^29^ and Antonić et al.^27^ Similar results were also described for *Abelmoschus esculentus* ^33^, *Sorghum bicolor* ^34^, and *Rosa hybrida* ^35^. Foliar application of 50 µM MeJA increased *I. walleriana* shoots FW and total leaf area at 15% SWC, while an increased shoots DW was observed at both drought intensities. Methyl jasmonate increased FW and DW of shoots and roots during salt-induced stress in *Brasica napus* ^36^, while similar results were described for *Brasica sp*. under polyethylene glycol-induced drought and exogenously applied MeJA^37^. Pre-treatment of 3-days-old wheat seedlings with 0.1 μM MeJA increased fresh weight and length of root and shoot during exposure to drought induced by different concentrations of mannitol^38^. Similar results considering different growth parameters were previously described in five wheat cultivars under exogenous application of MeJA seven days after drought induction^23^. Also, foliar application of MeJA (20 µM) increased the length, fresh, and dry shoot and root mass of *Glycine max* during dehydration^24^. Interestingly, the results of this research show no significant changes in *I. walleriana* shoots FW at 5% SWC under different drought treatment. However, shoots DW was increased in plants treated with both MeJA concentrations and then exposed to soil irrigation up to 5%. In general, it has been reported that DW can provide more precise measurement of biomass, eliminating fluctuations caused by water content in fresh tissues^39^. Therefore, it can be concluded that the foliar application of MeJA had a clearly positive effect on *I. walleriana* biomass under drought stress. It is very important to note that foliar application of MeJA affected the DMC of *I. walleriana* at both SWC, with the effect of 50 μM MeJA being greatest at 5% SWC. In addition, the increase in DMC at 5% SWC correlated with the changes in DW. Dry matter content is considered a measure of dry matter concentration in plants and could be a good indicator of variations in relative plant growth rate and resource utilization strategies^31;40^.

In recent years, nondestructive measurements of plant leaf parameters have been widely used in plant physiology related to photosynthesis^41–44^. Drought is a significant factor in impairing photosynthesis through decreasing CO_2_ availability and stomatal closure. Plants reduce stomatal conductance during drought, providing greater water retention in tissues, and increased cell turgidity. Reduced stomatal conductance during drought has been observed in *Vitis vinifera* ^45^, *Cicer arietinum* ^46^, *Oryza sativa* ^47^ and many other plant species. In our study, plants treated with a higher MeJA concentration at 15% SWC had slightly higher stomatal conductance, but without statistical significance, while at 5% SWC, stomatal conductance decreased in plants treated with both MeJA concentrations, compared with plants treated with ddH_2_O. The effect of MeJA on stomatal conductance reduction during drought has also been described in *T. aestivum* ^48^ and *Hordeum vulgare* ^49^, while in *B. oleracea* treated with MeJA stomatal conductance increased during drought, compared to control plants^50^. Both types of responses could be considered as plants adaptation to the drought conditions but also can be attributed to the plant genotype itself. The additional reduction of stomatal conductance by MeJA could reduce transpiration and water loss during drought, and could also affect the increment of *Water Use Efficiency* ^48^.

To investigate *I. walleriana* leaf physiology changes under drought and foliar applied MeJA, different physiological parameters, such as chlorophyll, flavonoids, anthocyanin, and NBI were measured. Our results showed no significant changes in all analyzed parameters in drought-stressed plants treated with ddH_2_O, relative to control plants. In the previous research, author Đurić et al.,^29^ described an increment in total chlorophyll and polyphenols in *I. walleriana* leaves during drought. Total chlorophyll and polyphenols were measured spectrophotometrically, and this increment is explained as an adaptive response of plants to drought. The main difference between this and mentioned research is the foliar treatment of drought-stressed plants with ddH_2_O in this research, and in the used measurement method. Foliar treatment with ddH_2_O may cause no changes in chlorophyll, anthocyanin, flavonoid, and NBI in drought-stressed *I. walleriana*, but could also be attributed to method sensitivity. Despite the changes in *I. walleriana* growth parameters, it could be concluded that the leaf physiology parameters measured by Dualex are not suitable for explaining the changes in plant productivity, namely, the changes in biomass during drought in plants foliar sprayed with ddH_2_O. However, nitrogen, as well as chlorophyll, play an important role in plant developmental processes and are closely related to the plant productivity. Nitrogen deficiency could occur due to water deficit and reduced absorption by the roots, as well as the inhibition of enzymes involved in N metabolism^51^. Since soil N is mainly controlled by microbial processes^52^, and microbial activity may still continue after soil drying^53^, this could be the explanation for unchanged N content in soil during drought, and therefore NBI in *I. walleriana* leaves. Consequently, unchanged N content and NBI may also result in unchanged chlorophyll content in plant leaves. It could be said that in this study a positive correlation between chlorophyll and NBI was observed in all applied treatments. Positively correlated changes in chlorophyll and NBI have also been described in wheat^43^. Significant changes in these parameters were observed only at 5% SWC, in *I. walleriana* plants foliar sprayed with a higher MeJA concentration (50 µM). Namely, plants foliar sprayed with 50 µM MeJA had higher Chlorophyll and NBI during intensive drought, indicating a positive elicitor effect on these parameters. This could be related to the MeJA effect on plant growth improvement under drought^23,24,54,55^. However, plants foliar sprayed with 50 µM MeJA had a decrease content of flavonoids in the leaf epidermis during intensive drought, which is also in correlation with increased NBI^12^. Nitrogen, as an essential macronutrient, has potential trade-off effects between growth and the secondary metabolism rate, so its increment could explain the reduced Flavonoid index in *I. walleriana* leaves.

## 5. Conclusion

In summary, our study shows that drought (especially 15% SWC) significantly reduced *I. walleriana* shoots FW and DW, as well as total leaf area, which could be improved by MeJA foliar application. However, Chlorophyll index and NBI did not restrict *I. walleriana* growth under drought, showing that water restriction impairs cell growth and elongation due to the loss of turgor is considered the most important limiting factor during drought. However, foliar application of 50 µM MeJA altered the physiological response of *I. walleriana’s* to drought and increased chlorophyll and NBI during intensive drought. In this way, photosynthesis could be improved, resulting in a higher matter of the *I. walleriana* shoot.

## Data Availability

Data available within the article or its supplementary materials.
